# Phylogenetic Divergence and Domestication Jointly Shape the Tomato Root Microbiome

**DOI:** 10.3390/plants15010163

**Published:** 2026-01-05

**Authors:** Grigorios Thomaidis, Georgios Boutzikas, Athanasios Alexopoulos, Christos Zamioudis

**Affiliations:** 1 Laboratory of Plant Pathology, Department of Agricultural Development, Democritus University of Thrace, 68200 Orestiada, Greece; grigthom1@agro.duth.gr (G.T.); gboutzik@agro.duth.gr (G.B.); 2 Laboratory of Microbiology, Biotechnology & Hygiene, Department of Agricultural Development, Democritus University of Thrace, 68200 Orestiada, Greece; alexopo@agro.duth.gr

**Keywords:** tomato domestication, wild relatives, root microbiome, rhizosphere, endosphere, 16S rRNA sequencing, Streptomycetaceae, Bacillaceae, nitrifying bacteria, breeding

## Abstract

Domestication reduced the genetic diversity in modern crops, often resulting in reduced resilience to biotic and abiotic stress. Evidence is now accumulating that domestication also altered the structure and function of root-associated microbiomes, creating new opportunities to harness beneficial microbes for breeding and crop improvement. Using multi-region 16S rRNA sequencing, we compared the rhizosphere and endosphere bacterial communities of cultivated tomato (*Solanum lycopersicum* cv. Moneymaker) with six wild relatives (*S. pimpinellifolium*, *S. huaylasense*, *S. peruvianum*, *S. chilense*, *S. habrochaites,* and *S. pennellii*) spanning the main wild lineages within *Solanum* sect. *Lycopersicon*. Bacterial community structure in the rhizosphere was broadly conserved across all seven hosts, and diversity remained comparable among genotypes. Despite this overall stability, the rhizosphere microbiomes were ordered along a gradient consistent with host phylogeny, with Moneymaker clustering near *S. pimpinellifolium*, the four green-fruited *Eriopersicon* species forming a cohesive block, and *S. pennellii* occupying the most distinct position. Within this hierarchy, individual hosts showed specific recruitment preferences, including enrichment of Streptomycetaceae in *S. pimpinellifolium*, Bacillaceae in *S. chilense*, and contrasting patterns of nitrifiers among *Eriopersicon* species and *S. pennellii*. Differential abundance testing in the endosphere revealed consistent reductions in several bacterial families in wild accessions, alongside the enrichment of Streptomycetaceae and Rhodobiaceae in multiple wild species. Overall, our study suggests that domestication exerted a modest effect on tomato root microbiomes, while wild relatives retained microbial association traits that could be harnessed in microbiome-informed breeding to improve resilience in cultivated tomato.

## 1. Introduction

Crop domestication allowed humans to select for desirable agronomic characteristics and marked a major turning point in human history. However, this artificial selection, further amplified by plant breeding practices, has left most contemporary cultivars with markedly reduced genetic diversity compared to their wild relatives [1,2,3]. As a consequence, modern varieties and widely used hybrids may express reduced resilience in several respects, for instance, showing lower resistance to certain pathogens and pests or limited tolerance to abiotic stresses [4]. Wild relatives remain therefore an indispensable resource for crop breeding, providing access to various useful traits that have been diminished or lost during domestication [5,6].

In the soil directly surrounding plant roots (rhizosphere) and within root tissues (endosphere), plants host diverse microbial communities that together form a functional microbiome. These root-colonizing microbial assemblages contribute to plant productivity by promoting growth, improving nutrition and enhancing protection against pathogens [7,8]. Comparative studies across several major crops, including maize [9], wheat [10,11], rice [12], and common bean [13], have revealed alteration in both the rhizosphere and endophytic root microbiomes between wild progenitors and domesticated hosts, often involving changes in microbial functional groups associated with nutrient cycling and stress tolerance [14,15]. Nevertheless, the magnitude and consistency of domestication effects are still being clarified, particularly as microbiome-informed crop breeding emerges as a promising direction for improving resilience in modern cultivars [16].

Tomato (*Solanum lycopersicum*) presents a well-characterized domestication history with marked reductions in genetic variability relative to its wild relatives [17]. Previous studies have shown that cultivated tomatoes and wild relatives differ in their microbiomes [18,19,20]. However, how belowground microbial communities diverge along the domestication continuum remains poorly resolved. Here, using multi-region 16S rRNA sequencing, we profiled the rhizosphere and root endosphere microbiomes of *S. lycopersicum* cv. Moneymaker and six wild relatives belonging to the *Lycopersicon* group (represented here by *S. pimpinellifolium*), the phylogenetically intermediate *Eriopersicon* group (represented here by *S. huaylasense*, *S. peruvianum*, *S. chilense* and *S. habrochaites*), and the more divergent *Neolycopersicon* group (represented here by *S. pennellii*) [21,22,23,24]. Within this phylogenetic framework, we hypothesized that genetically related hosts would harbor more similar microbiomes, such that the microbiome of the cultivated tomato would be most similar to its closest wild relative, *S. pimpinellifolium*, and increasingly distinct in more distantly related wild species. Beyond this general pattern, individual hosts are expected to display unique microbiome features, particularly in the root endosphere where host control over microbial communities is stronger. By integrating community composition analyses with differential abundance modeling, we evaluated these hypotheses while establishing a comparative framework relevant to microbiome-informed breeding strategies in tomato.

## 2. Results

### 2.1. Sequencing Output and Dataset Overview

A total of 45 microbiome samples were successfully sequenced, representing bulk soil, rhizosphere and root endosphere compartments across seven tomato genotypes (one cultivated and six wild relatives), each sampled in biological triplicate. Sequencing of 16S rRNA gene amplicons targeting six hypervariable regions (V2, V3, V4, V6–7, V8, and V9) produced 14,219,208 reads. Following the removal of low-quality or short reads and sequences not assignable to reference databases, 6,012,581 high-confidence reads remained and were used for taxonomic classification and downstream analyses. Rarefaction curves indicated sufficient sequencing depth across all samples (Supplementary Figure S1).

### 2.2. Phylum-Level Microbiome Profiles in the Domesticated Host and the Wild Relatives

**Figure 1:**
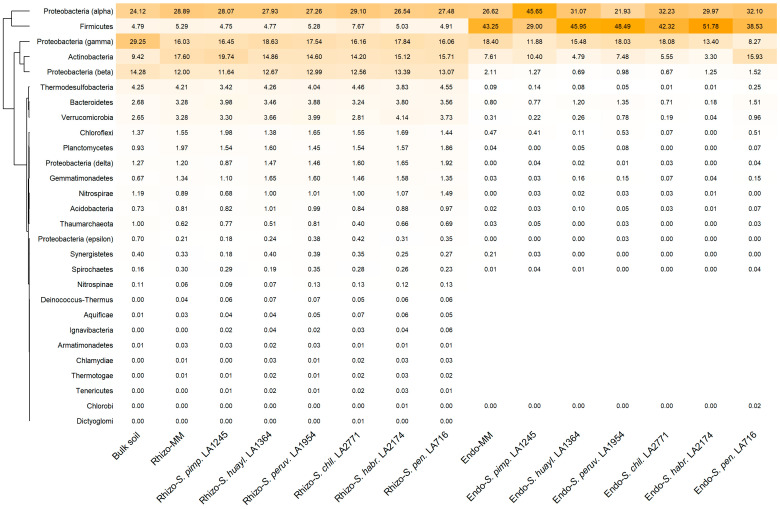
Heatmap showing the mean % relative abundance of major bacterial phyla in the bulk soil, the rhizosphere (Rhizo-) and endosphere (Endo-) of the cultivated tomato *Solanum lycopersicum* cv. Moneymaker (MM) and the wild relatives *S. pimpinellifolium* (*S. pimp.* LA1245), *S. huaylasense* (*S. huayl.* LA1364), *S. peruvianum* (*S. peruv.* LA1954), *S. chilense* (*S. chil.* LA2771), *S. habrochaites* (*S. habr.* LA2174), and *S. pennellii* (*S. pen.* LA716). Values shown as 0.00 represent phyla with <0.01% relative abundance; blank cells correspond to taxa not detected in the respective samples.

In the bulk soil, bacterial communities were dominated by Proteobacteria, which together accounted for roughly 70% of the total relative abundance, with Gammaproteobacteria, Alphaproteobacteria, and Betaproteobacteria as the major contributing classes. Actinobacteria represented an additional substantial fraction, followed by Firmicutes, Thermodesulfobacteria, Bacteroidetes, and Verrucomicrobia. Other phyla occurred at lower abundances (Figure 1). In the rhizosphere, this overall phylum-level community structure was largely conserved across all seven hosts. However, two consistent shifts related to bulk soil were evident: a decrease in Gammaproteobacteria and an enrichment in Actinobacteria. These trends were shared by both the cultivated tomato and the wild relatives (Figure 1).

The endosphere displayed a distinct microbiome dominated by Firmicutes, Alphaproteobacteria, Gammaproteobacteria, and Actinobacteria, which together constituted more than 70% of the total community across all genotypes. Alphaproteobacteria and Firmicutes were particularly abundant and showed little variability among plant genotypes. Gammaproteobacteria and Actinobacteria varied more markedly among hosts, while other phyla occurred at low or sporadic abundance (Figure 1).

### 2.3. Family-Level Microbiome Composition Shows Patterns Consistent with Host Phylogeny

**Figure 2:**
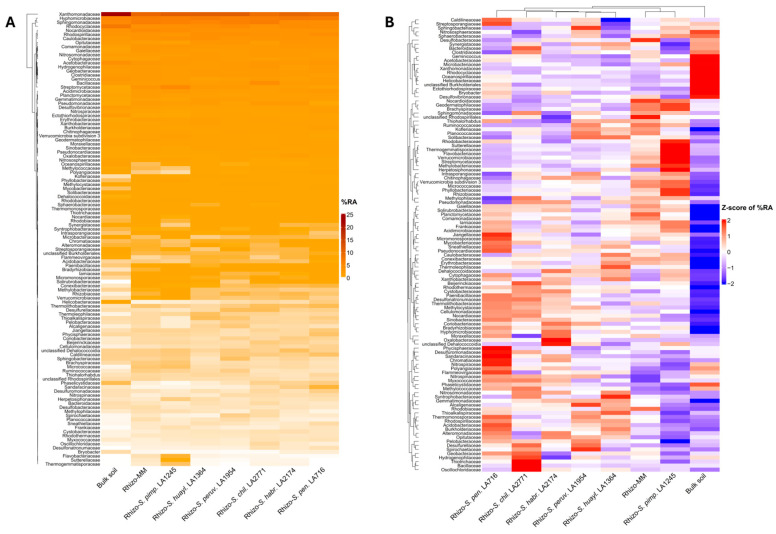
(**A**) Heatmap showing the mean % relative abundance of major bacterial families in the bulk soil and the rhizosphere (Rhizo-) of the cultivated tomato *S. lycopersicum* cv. Moneymaker (MM) and the wild relatives *S. pimpinellifolium* (*S. pimp.* LA1245), *S. huaylasense* (*S. huayl.* LA1364), *S. peruvianum* (*S. peruv.* LA1954), *S. chilense* (*S. chil.* LA2771), *S. habrochaites* (*S. habr.* LA2174), and *S. pennellii* (*S. pen.* LA716). Only families with a mean relative abundance ≥ 0.1% in at least one experimental group are shown. (**B**) Z-score-scaled heatmap of the same bacterial families shown in (**A**), highlighting relative enrichment and depletion across genotypes. Z-scores were calculated by centering and scaling percentage relative abundance values for each bacterial family across genotypes; red indicates relative enrichment and blue relative depletion. Hierarchical clustering was applied using Euclidean distance and complete linkage.

To resolve finer-scale taxonomy in the rhizosphere and endosphere microbiomes, we next examined community structure at the family level. Consistent with the phylum-level profiles, bacterial communities in the rhizosphere of all genotypes broadly reflected those of the surrounding bulk soil, indicating that most dominant soil families persisted in the root vicinity. Nevertheless, several bacterial families appeared enriched or depleted relative to bulk soil, reflecting the selective influence of roots on nearby microbes (Figure 2A). Z-scaled hierarchical clustering separated bulk soil from all rhizosphere samples; *S. lycopersicum* cv. Moneymaker (hereafter Moneymaker) clustered with *S. pimpinellifolium*, while the remaining wild species followed a compositional gradient: *S. huaylasense* and *S. peruvianum* grouped together, followed by *S. habrochaites*, *S. chilense*, and *S. pennellii* at the most distant position (Figure 2B). This ordering provides an initial indication that rhizosphere microbiome composition is structured in relation to host phylogeny.

**Figure 3:**
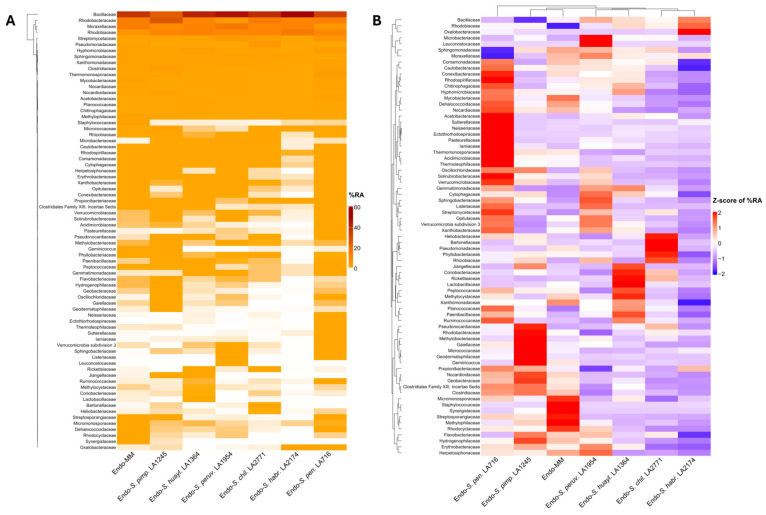
(**A**) Heatmap showing the mean % relative abundance of major bacterial families in the endosphere (Endo-) of the cultivated tomato *S. lycopersicum* cv. Moneymaker (MM) and the wild relatives *S. pimpinellifolium* (*S. pimp.* LA1245), *S. huaylasense* (*S. huayl.* LA1364), *S. peruvianum* (*S. peruv.* LA1954), *S. chilense* (*S. chil.* LA2771), *S. habrochaites* (*S. habr.* LA2174), and *S. pennellii* (*S. pen.* LA716). Only families with a mean relative abundance ≥ 0.1% in at least one experimental group are shown. (**B**) Z-score-scaled heatmap of the same bacterial families shown in (**A**), highlighting relative enrichment and depletion across genotypes. Z-scores were calculated by centering and scaling percentage relative abundance values for each bacterial family across genotypes; red indicates relative enrichment and blue relative depletion. Hierarchical clustering was applied using Euclidean distance and complete linkage.

In the root interior, Bacillaceae, Moraxellaceae, Rhodobacteraceae, and Rhodobiaceae dominated across genotypes, jointly exceeding 70% relative abundance and reaching almost 90% in *S. habrochaites* (Figure 3A). Z-scaled clustering revealed a compact pairing of Moneymaker with *S. pimpinellifolium*, while *S. peruvianum* grouped with *S. huaylasense* and *S. chilense* clustered with *S. habrochaites* within the same branch (Figure 3Β). Thus, unlike the rhizosphere gradient, the endosphere resolved into paired lineage blocks, indicating lineage-associated groupings shaped by stronger internal filtering.

### 2.4. Microbial Alpha Diversity Is Strongly Affected by Compartment but Shows Minor Differences Among Genotypes

**Figure 4:**
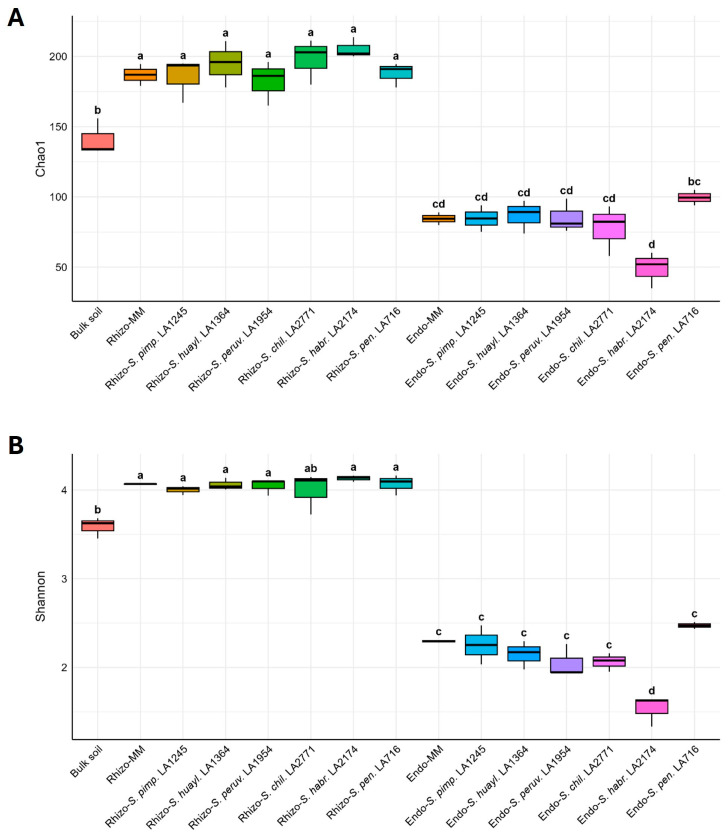
Microbial alpha diversity in the rhizosphere (Rhizo-) and root endosphere (Endo-) of the cultivated tomato *S. lycopersicum* cv. Moneymaker (MM) and its wild relatives *S. pimpinellifolium* (*S. pimp.* LA1245), *S. huaylasense* (*S. huayl.* LA1364), *S. peruvianum* (*S. peruv.* LA1954), *S. chilense* (*S. chil.* LA2771), *S. habrochaites* (*S. habr.* LA2174), and *S. pennellii* (*S. pen.* LA716). (**A**) Chao1 richness index. (**B**) Shannon diversity index. Letters indicate statistical groupings according to Tukey’s HSD post hoc test (*p* < 0.05).

To assess differences in microbial richness and evenness among genotypes, we calculated Chao1 and Shannon alpha diversity indices (Figure 4). Both metrics satisfied assumptions of normality and homogeneity of variances (Shapiro–Wilk *p* > 0.05; Levene’s *p* > 0.7), and ANOVA indicated significant differences for both metrics (*p* < 0.001). Alpha diversity in the rhizosphere was significantly higher than in bulk soil for both the Chao1 and Shannon indices, with the exception of *S. peruvianum*, for which the Shannon diversity did not differ significantly between the rhizosphere and bulk soil; no statistically significant differences in alpha diversity were observed among tomato genotypes (Figure 4A,B).

Microbial communities in the endosphere showed lower diversity than in the rhizosphere and bulk soil. Most genotype comparisons were not significant, although *S. pennellii* exhibited higher richness and Shannon diversity than *S. habrochaites*. Overall, *S. pennellii* hosted the most diverse endosphere microbiome, whereas *S. habrochaites* exhibited the lowest diversity, with a significantly reduced Shannon index relative to all other genotypes (Figure 4A,B).

### 2.5. Beta-Diversity Reveals Host-Phylogeny-Aligned Structuring of Rhizosphere and Endosphere Microbiomes

**Figure 5:**
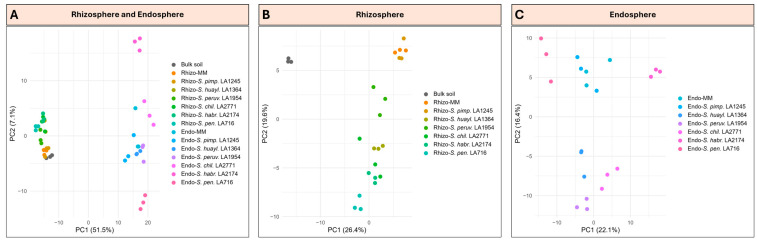
Principal component analysis (PCA) of bacterial community composition based on Aitchison distances. (**A**) Combined ordination of bulk soil, rhizosphere (Rhizo-) and endosphere (Endo-) samples of the cultivated tomato *Solanum lycopersicum* cv. Moneymaker (MM) and the wild relatives *S. pimpinellifolium* (*S. pimp.* LA1245), *S. huaylasense* (*S. huayl.* LA1364), *S. peruvianum* (*S. peruv.* LA1954), *S. chilense* (*S. chil.* LA2771), *S. habrochaites* (*S. habr.* LA2174), and *S. pennellii* (*S. pen.* LA716). (**B**) PCA of bulk soil and rhizosphere samples for the same genotypes. (**C**) PCA of root endosphere samples for the same genotypes. PERMANOVA: compartment effect (*F* = 16.16, *p* = 0.001); rhizosphere genotypes (*F* = 5.86, *p* = 0.001); endosphere genotypes (*F* = 5.53, *p* = 0.001).

Building on the z-scaled clustering patterns, we quantified microbiome differentiation more explicitly by computing Aitchison distances on CLR-transformed data and visualizing these via PCA (Figure 5). In the combined PCA (bulk soil, rhizosphere and endosphere samples), the ordination revealed clear compartmental separation: rhizosphere and endosphere microbiomes formed two well-resolved clusters, while bacterial communities in the bulk soil remained adjacent but distinct from plant-associated communities (Figure 5A). A global PERMANOVA confirmed a dominant compartment effect (*F* = 26.254, *p* = 0.001).

In the rhizosphere PCA (bulk soil and rhizosphere samples only), bulk soil resolved distinctly from all plant-associated microbiomes, reflecting a strong shift away from the soil baseline. Rhizosphere microbiomes were positioned along a directional gradient that reflected host–phylogenetic relatedness, with Moneymaker clustering with *S. pimpinellifolium*, the *Eriopersicon* species following consecutively, and *S. pennellii* defining the most distant endpoint (Figure 5B). PERMANOVA supported significant genotype effects (*F* = 7.539, *p* = 0.001), matching the ordering previously observed in z-scaled clustering and confirming that rhizosphere microbiome composition reflects host–phylogenetic distance.

In the endosphere PCA (endosphere samples only), microbial communities formed discrete and compact lineage-associated clusters, rather than a continuous gradient. Moneymaker and *S. pimpinellifolium* harbored closely related communities, forming a tight pair. A second cluster grouped *S. peruvianum*, *S. huaylasense* and *S. chilense* at a separate position from the Moneymaker–*S. pimpinellifolium* pair. In contrast, *S. pennellii* and *S. habrochaites* each formed isolated clusters, reflecting distinct endophytic assemblages (Figure 5C). PERMANOVA confirmed significant host effects on endosphere composition (*F* = 7.295, *p* = 0.001). Thus, beta diversity analysis indicates that the endosphere microbiome retains a host–phylogenetic signal, expressed not as a continuous gradient but as distinct lineage-defined clusters.

### 2.6. Differentially Abundant Bacterial Families Between Cultivated Tomato and Wild Relatives

To identify the bacterial families driving variation in microbiome composition across genotypes, we performed MaAsLin2 differential abundance analysis across three contrasts: (i) each rhizosphere versus bulk soil, (ii) rhizospheres of the wild relatives versus Moneymaker, and (iii) endospheres of the wild relatives versus Moneymaker. Taxa with q < 0.1 were considered differentially abundant (DA) (Supplementary Figure S2).

**Figure 6:**
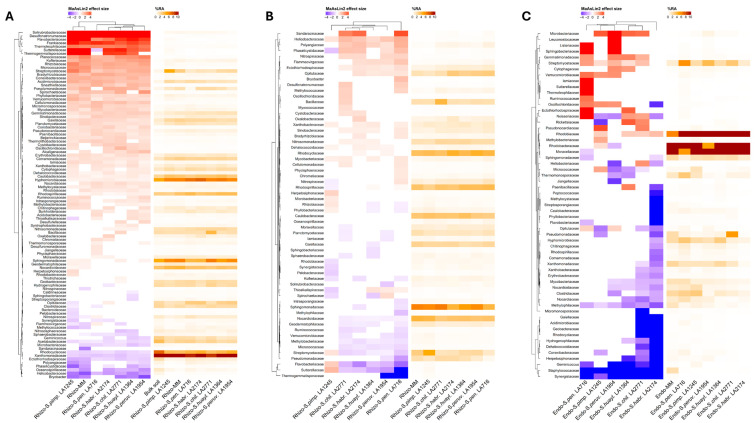
Heatmap of differentially abundant (DA) bacterial families identified by MaAsLin2. (**A**) DA families in the rhizosphere of each genotype compared to bulk soil. (**B**) DA families in the rhizosphere of the wild relatives compared to the Moneymaker rhizosphere. (**C**) DA families in the endosphere of the wild relatives compared to the Moneymaker endosphere. MaAsLin2 results are shown as log_2_ fold change effect sizes (blue = depletion, red = enrichment). Mean relative abundance (%RA) is displayed separately to contextualize taxon prevalence independent of effect-size estimates. Only families with mean %RA ≥ 0.1% in at least one genotype were included in the models and only features with q < 0.1 in at least one contrast are displayed. Hierarchical clustering was performed on effect-size matrices (Euclidean distance, complete linkage).

Ιn the rhizosphere, comparisons against bulk soil revealed a largely conserved recruitment pattern across hosts: several low-abundance soil families were significantly enriched at the root interface, whereas many dominant soil taxa declined in relative abundance (Figure 6A and Figure S2). This conserved set of differentially abundant families provides a taxonomic basis for the clear separation between bulk soil and rhizosphere communities observed in the PCA.

**Figure 7:**
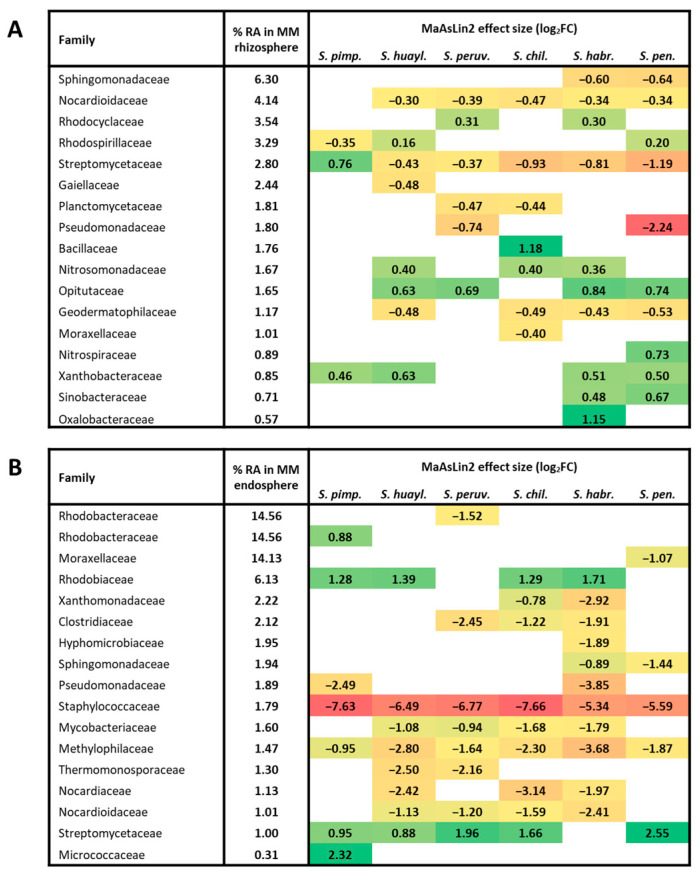
A subset of differentially abundant (DA) bacterial families (q < 0.1) with ≥ 1% mean relative abundance in at least one genotype. For these families, MaAsLin2 results are shown as log_2_ fold change effect sizes for each wild accession relative to *S. lycopersicum* cv. Moneymaker (MM). (**A**) Rhizosphere. (**B**) Endosphere. Positive coefficients (green) indicate enrichment in wild genotypes, while negative values (yellow-red) indicate depletion relative to MM. Mean % relative abundance in MM is shown for each plot.

When the rhizosphere of each of the wild accessions was compared directly with that of Moneymaker, only a limited number of families showed significant changes, indicating that genotype-specific deviations from the cultivated reference were modest. Consistent with the rhizosphere PCA, *S. pimpinellifolium* exhibited the smallest number of differentially abundant families and generally mild effect sizes, whereas more distant wild species showed both a higher number of differentially abundant families and stronger abundance shifts (Figure 6B and Figure S2). Importantly, several of the families contributing to this pattern correspond to abundant components of the rhizosphere microbiome across hosts (≥1% relative abundance in at least one genotype) (Figure 7A). Among these abundant families, several functionally relevant patterns emerged: Streptomycetaceae were selectively enriched in *S. pimpinellifolium*, Bacillaceae in *S. chilense*, whereas *S. pennellii* showed reduced Streptomycetaceae and Pseudomonadaceae but a marked increase in Nitrospiraceae. Nitrosomonadaceae were higher across multiple *Eriopersicon* species, indicating divergent nitrogen cycling capacity along the wild lineage (Figure 7A). Overall, despite broad conservation in rhizosphere community structure, host-specific microbial recruitment persists.

Compared to the rhizosphere, DA families in the endosphere differed more strongly among the genotypes, and this pattern is reflected in the discrete, lineage-associated separation observed in the PCA (Figure 6C and Figure S2). Notably, several DA families exhibiting strong abundance differences corresponded to abundant components of the endosphere microbiome (≥1% relative abundance in at least one genotype) (Figure 7B), suggesting that variation in these families contributes substantially to the observed PCA separation. Among these abundant families, Staphylococcaceae, Methylophilaceae, Mycobacteriaceae, Nocardiaceae, and Nocardioidaceae were consistently depleted in the endosphere of most wild relatives relative to Moneymaker. Conversely, Streptomycetaceae and Rhodobiaceae were recurrently enriched, particularly in *S. pennellii*, which displayed the strongest restructuring of internal communities. Additional genotype-associated shifts included lower Xanthomonadaceae in *S. chilense* and *S. habrochaites* and elevated Micrococcaceae in *S. pimpinellifolium* (Figure 7B).

## 3. Discussion

In this study, we investigated how domestication and host phylogeny jointly influence the assembly of rhizosphere and endosphere microbiomes by comparing a cultivated tomato with selected wild relatives spanning the main evolutionary lineages of *Solanum* sect. *Lycopersicon*. Our results indicate that these processes resulted in distinct, compartment-dependent signatures, with domestication sensu stricto (cultivated versus wild) being associated with rather modest shifts that were most evident in the rhizosphere, whereas stronger and more heterogeneous differences arose among wild lineages, particularly within the root endosphere.

In the rhizosphere, alpha diversity indices were broadly similar across all hosts, consistent with reports that domestication does not necessarily reduce rhizosphere diversity [25,26]. Across all seven genotypes, the rhizosphere maintained a broadly conserved recruitment pattern, indicating that domestication has not fundamentally altered the dominant features of belowground microbial assembly. This observation aligns with broader evidence that domestication modifies but does not eliminate core rhizosphere filtering processes [27,28]. Within this conserved baseline, rhizosphere microbiomes followed a clear host-lineage-aligned structure: the cultivated tomato clustered with *S. pimpinellifolium*, its closest wild relative, the four green-fruited *Eriopersicon* species formed a different group, and *S. pennellii* occupied the most divergent position. The close similarity between the cultivated tomato and *S. pimpinellifolium* suggests limited domestication-driven divergence in the rhizosphere, whereas the broader separation among wild species is better explained by deeper evolutionary differentiation within the section. These patterns are consistent with previous studies demonstrating that host genetic background and domestication history are major determinants of rhizosphere community structure [10,25,29].

While the rhizosphere microbiome remains broadly conserved across tomato genotypes, individual wild relatives exhibit distinct recruitment fingerprints involving specific bacterial families. Several of these families are frequently discussed in the literature in relation to particular functional traits. Streptomycetaceae—rich in antibiotic-producing and plant-beneficial taxa [30]—were selectively enriched in *S. pimpinellifolium*, while Bacillaceae, a family frequently linked to stress tolerance and growth promotion [31], increased specifically in *S. chilense*. Nitrifying bacteria also diverged across lineages, with Nitrosomonadaceae elevated in multiple *Eriopersicon* species and Nitrospiraceae markedly enriched in *S. pennellii*, suggesting differences in nitrogen transformation potential [32]. Importantly, these functional interpretations are inferred from taxonomic composition rather than directly measured activity, and several of the families discussed encompass substantial ecological and functional heterogeneity. Consequently, family-level enrichment may mask divergent or even opposing trends at finer taxonomic resolution. The patterns reported here should therefore be interpreted as reflecting broad lineage-level tendencies rather than uniform functional responses across all constituent taxa, a limitation inherent to the family-level analytical resolution imposed by the multi-region Ion Torrent 16S rRNA sequencing approach.

In contrast to the rhizosphere, the endosphere microbiome exhibited stronger host-specific filtering and more pronounced divergence among genotypes while showing a weaker and less uniform relationship with host genetic relatedness, consistent with evidence from other crops suggesting that internal root microbiomes are largely governed by genotype-specific physiological and immunological traits [33,34]. Wild species diverged from Moneymaker in heterogeneous ways: some, such as *S. habrochaites*, exhibited broad reductions across multiple families, whereas others—notably *S. pennellii*—retained more diverse and selectively enriched endophytic assemblages. Several abundant families, including Staphylococcaceae, Methylophilaceae, Mycobacteriaceae, Nocardiaceae, and Nocardioidaceae, were consistently depleted in the endosphere of most wild relatives, suggesting a shared tendency to restrict taxa that commonly behave as generalist or opportunistic root colonizers. Such depletion could be attributed to stronger host-mediated filtering of internal tissues, a process that has been linked to plant immune regulation, root structural traits, and physical barriers. In tomato, domestication has been associated with changes in immune- and defense-related pathways, including reduced salicylic acid and phenolamide biosynthesis, metabolites known to influence endosphere colonization and the internal establishment of specific bacterial taxa [33,35,36,37]. Differences between cultivated and wild tomatoes in root system architecture may further modulate microbial entry into internal tissues by altering the number and spatial distribution of entry points from the rhizosphere [8,13]. In addition, physical barrier traits, such as the formation of a suberized exodermis, have been shown to restrict diffusion and microbial access beyond the rhizoplane in tomato, with variation in such barriers influencing internal colonization [38,39,40]. Conversely, Streptomycetaceae and Rhodobiaceae were enriched in multiple wild relatives, groups often associated with specialized metabolic capabilities, including antibiotic production [41,42]. Overall, these patterns indicate that endosphere microbiomes exhibit sharper, genotype-dependent shifts than those observed in the rhizosphere, reflecting differential host control over internal microbial colonization rather than domestication sensu stricto.

Several aspects of our findings point toward practical opportunities for microbiome-informed breeding. The genotype-specific enrichment of potentially functionally important bacterial groups such as Streptomycetaceae in *S. pimpinellifolium*, Bacillaceae in *S. chilense*, and nitrifiers in the *Eriopersicon* species and *S. pennellii* suggests that wild tomatoes retain heritable traits capable of modulating microbial recruitment. These traits, which may relate to exudation chemistry, root immunity, or nutrient acquisition, provide tractable biological targets for breeding efforts aiming to enhance associations with beneficial taxa [43]. Such trait-linked microbial signatures can also guide QTL mapping to identify host loci influencing the abundance of key functional families and facilitate their introgression into elite backgrounds [44,45]. In a broader comparative context, similar patterns have been reported in other cultivated plant systems, indicating that the trends observed here are not unique to tomato: studies in maize, common bean, and cereals show that domestication and host genetic variation exert modest but detectable effects on root-associated microbiomes, while soil and environmental factors remain dominant drivers of overall community composition. Across these systems, host effects tend to be more pronounced in internal root compartments than in the rhizosphere, paralleling the compartment-specific patterns observed in the present study [14,27,46,47].

Several limitations in our study should be acknowledged. Plants were grown in a heterogeneous soil-based substrate rather than native field soil, which may influence microbial recruitment dynamics and limit direct ecological extrapolation. Nevertheless, field-based tomato microbiome studies indicate that host genotype and host lineage retain a detectable influence on root-associated microbial communities across diverse soil types and environmental conditions, including native habitats of wild tomatoes [48,49,50]. In addition, the multi-region Ion Torrent 16S approach increases taxonomic breadth relative to single-amplicon sequencing, yet family-level aggregation, although reducing noise, masks strain-level variation with potential functional relevance. Future work combining shotgun metagenomics, metatranscriptomics, or targeted functional assays will therefore be required to determine whether the observed lineage-specific taxonomic shifts translate into functional differences. Such approaches would enable more direct linkage between host genotype, microbial gene content, and functional expression, refining the ecological and agronomic interpretation of the compositional patterns reported here. Finally, in this study, the moderate level of replication may have constrained our ability to detect more subtle host-driven effects. Despite these considerations, our findings converge on a coherent pattern in which domestication-related effects are primarily expressed as subtle modifications of an otherwise conserved rhizosphere microbiome, whereas deeper evolutionary divergence among wild tomato lineages drives stronger, genotype-specific restructuring in the root endosphere. These contrasting architectures suggest that wild tomatoes preserve microbial recruitment traits valuable for selection, positioning them as reservoirs for microbiome-informed breeding toward enhanced resilience and function.

## 4. Materials and Methods

### 4.1. Plant Material and Microbiome Sampling

Microbiome samples were collected from the tomato cultivar *Solanum lycopersicum* cv. Moneymaker and six wild relatives, including *S. pimpinellifolium* LA1245, *S. huaylasense* LA1364, *S. peruvianum* LA1954, *S. chilense* LA2771, *S. habrochaites* LA2174, and *S. pennellii* LA716. Moneymaker seeds were sourced from a local supplier, and seeds of the wild species were obtained from the C.M. Rick Tomato Genetics Resource Center (TGRC). Uniform 12-day-old seedlings were transplanted into 2 L plastic pots filled with a soil mixture composed of four parts sieved agricultural soil collected from a fallow field, two parts commercial potting soil (Potgrond P; Klasmann-Deilmann GmbH, Geeste, Germany), two parts sieved sand from the Evros (Maritsa) river, and one part perlite. Plants were cultivated in a greenhouse facility under controlled conditions (natural photoperiod of approximately 12 h light/12 h dark; day/night temperatures averaging approximately 20–22 °C and 26–28 °C, respectively), irrigated as needed, and fertilized once per week with a modified half-strength Hoagland nutrient solution [51].

Rhizosphere samples were collected five weeks after transplantation by gently uprooting plants and removing loosely attached soil. The soil fraction tightly adhering to the root surface was recovered as the rhizosphere. For endosphere sampling, root systems were thoroughly washed and surface-sterilized following McKinnon [52]: 85% ethanol for 1 min; 30 s rinse in sterile distilled water (sdH_2_O); 2.5% sodium hypochlorite (NaOCl) for 5 min; and four successive 1 min washes in sdH_2_O. Bulk soil samples were obtained from unplanted control pots containing the same soil mix. All sample types were collected in biological triplicates, with each sample composed of five subsamples, yielding a total of 45 samples. Immediately after collection, samples were flash-frozen in liquid nitrogen and stored at −80 °C until further processing.

### 4.2. DNA Extraction, 16S rRNA Gene Amplification, Library Preparation, and Sequencing

Genomic DNA was extracted from the bulk soil, rhizosphere, and endosphere samples using the ZymoBIOMICS™ DNA Miniprep Kit D4300 (Zymo Research, Irvine, CA, USA), following the manufacturer’s protocol. DNA concentration and purity were assessed using a NanoPhotometer™ P-Class P330 (IMPLEN GmbH, Munich, Germany). DNA samples were stored at −20 °C until further processing. The 16S rRNA gene was amplified using the Ion 16S™ Metagenomics Kit (Thermo Fisher Scientific, Waltham, MA, USA), targeting regions V2, V3, V4, V6–7, V8, and V9. Each sample underwent two multiplex PCR reactions (V2-V4-V8; V3-V6–7-V9) under the following conditions: 95 °C for 5 min; 5 cycles of 95 °C for 15 s, 58 °C for 15 s, 70 °C for 1 min; 70 °C for 5 min. Combined PCR products were purified with AMPure XP beads (Beckman Coulter, Brea, CA, USA).

Genomic DNA libraries were prepared using the Ion Plus Fragment Library Kit in combination with the Ion Xpress™ Barcode Adapters Kit 1-96, following the manufacturer’s protocols (Thermo Fisher Scientific). Library quantification was performed with the Ion Universal Library Quantitation Kit (Thermo Fisher Scientific). Amplicons of approximately 400 bp were sequenced using the Ion 510™, Ion 520™, and Ion 530™ Kit-Chef reagents on an Ion Torrent S5XL platform (Thermo Fisher Scientific). Sequencing was carried out at CeMIA SA (Larissa, Greece).

### 4.3. Processing of Sequencing Data and Taxonomic Profiling

Raw sequence data (BAM files) were processed using Ion Reporter™ Software v5.2 with Metagenomics 16S Workflow v1.1. Reads were primer-trimmed and quality-filtered, and retained only if they were >150 bp, had ≥90% alignment coverage, and were represented by at least 10 reads per taxon. Taxonomic assignments were generated using the MicroSEQ^®^ 16S Reference Library (v2013.1) and Greengenes. Abundance tables (phylum to species) were exported for each hypervariable region and as consensus profiles; consensus data were used for all subsequent analyses. Downstream analyses were deliberately conducted at the family level, as the multi-region Ion Torrent 16S rRNA sequencing approach yields heterogeneous taxonomic resolution across hypervariable regions, thereby necessitating a consistent analytical framework across samples. To assess adequate sequencing depth, rarefaction curves were generated using the rarecurve() function from the vegan package (v2.7.2) [53] in R (v4.4.2). An additional downstream filtering step was applied to retain only those taxa with at least 10 read counts in at least two biological replicates within any experimental group. Descriptive phylum- and family-level taxonomic profiles were visualized using relative abundance heatmaps generated with the ComplexHeatmap R package (v2.22.0), with hierarchical clustering of taxa and samples. Differential abundance testing was conducted exclusively at the family level using MaAsLin2 (v1.10.0), as described below.

### 4.4. Alpha and Beta Diversity Analyses

Alpha and beta diversity analyses were performed at the family level in R using functions from the vegan package (v2.7.2) for rarefaction, diversity metrics, and PERMANOVA [53]. For alpha diversity, read counts were first rarefied to the minimum library size using rrarefy() to account for differences in sequencing depth. Chao1 richness and Shannon diversity were calculated with estimateR() and diversity(), respectively. Normality and homogeneity of variances were evaluated with Shapiro–Wilk and Levene’s tests, respectively. Differences among genotypes were assessed with one-way ANOVA, followed by Tukey’s HSD for pairwise comparisons, and results were visualized using ggplot2 (v4.0.0) boxplots with significance groups based on Tukey-adjusted *p*-values.

Beta diversity was quantified using Aitchison distances, computed as Euclidean distances on CLR-transformed abundance data. Distance matrices were generated with dist() in R (stats package, v4.4.2), and multivariate patterns were explored using PCA on CLR-transformed data with visualization in ggplot2. Community differences were tested using PERMANOVA via adonis2() (vegan package v2.7.2) [53]. Single-factor models were applied to rhizosphere and endosphere datasets, while a multifactor model evaluated compartment, genotype, and their interaction when datasets were combined.

### 4.5. Differential Abundance Analysis

Differential abundance analyses were performed using MaAsLin2 (v1.10.0) on family-level total-sum-scaled relative abundance data [54]. Input tables were filtered upstream to retain taxa with ≥0.1% mean relative abundance in at least one group. Within MaAsLin2, features present in <20% of samples were excluded (min_prevalence = 0.20), while no additional abundance filtering was applied (min_abundance = −Inf). Models were fitted using the linear model (LM) framework with no normalization (normalization = NONE) and no internal standardization (standardize = FALSE). A log transformation was applied prior to model fitting. Multiple testing correction was performed using the Benjamini–Hochberg false discovery rate (FDR) method (correction = BH). For each comparison, the reference level of the grouping variable was set according to the biological contrast of interest (Bulk soil, Moneymaker rhizosphere, Moneymaker endosphere). MaAsLin2 coefficients were interpreted as log_2_ fold changes relative to the reference group. Taxa with q < 0.1 were considered significantly differentially abundant. Heatmaps of MaAsLin2 effect sizes (log_2_ fold changes) together with corresponding relative abundance values, were generated using the ComplexHeatmap package (v2.22.0) in R, with hierarchical clustering applied to the taxa and samples.

## Data Availability

Raw 16S rRNA amplicon sequencing reads have been deposited in the NCBI Sequence Read Archive (SRA) under BioProject PRJNA1376498, with Run Accessions SRR36367991–SRR36368035.

## Supplementary Materials

The following supporting information can be downloaded at: https://www.mdpi.com/article/10.3390/plants15010163/s1. Figure S1. Rarefaction curves for bulk soil, rhizosphere, and endosphere samples, showing the number of observed families plotted against sequencing depth. Figure S2. Number of differentially abundant (DA) bacterial families identified by MaAsLin2 using a significance threshold of q < 0.1. (A) Rhizosphere of each genotype versus bulk soil. (B) Rhizosphere of each wild relative versus Moneymaker rhizosphere. (C) Endosphere of each wild relative versus Moneymaker endosphere. Numbers within bars indicate the total DA families per category.
