# Supplementary material for: Phylogenetic Divergence and Domestication Jointly Shape the Tomato Root Microbiome
Source: Plants (Basel). 2026 Jan 5;15(1):163. doi: 10.3390/plants15010163 (PMC12787688; doi:10.3390/plants15010163)
Supplement: Supplementary file 1 [file plants-15-00163-s001.zip › plants-4063617-supplementary.pdf]

Supplementary Materials

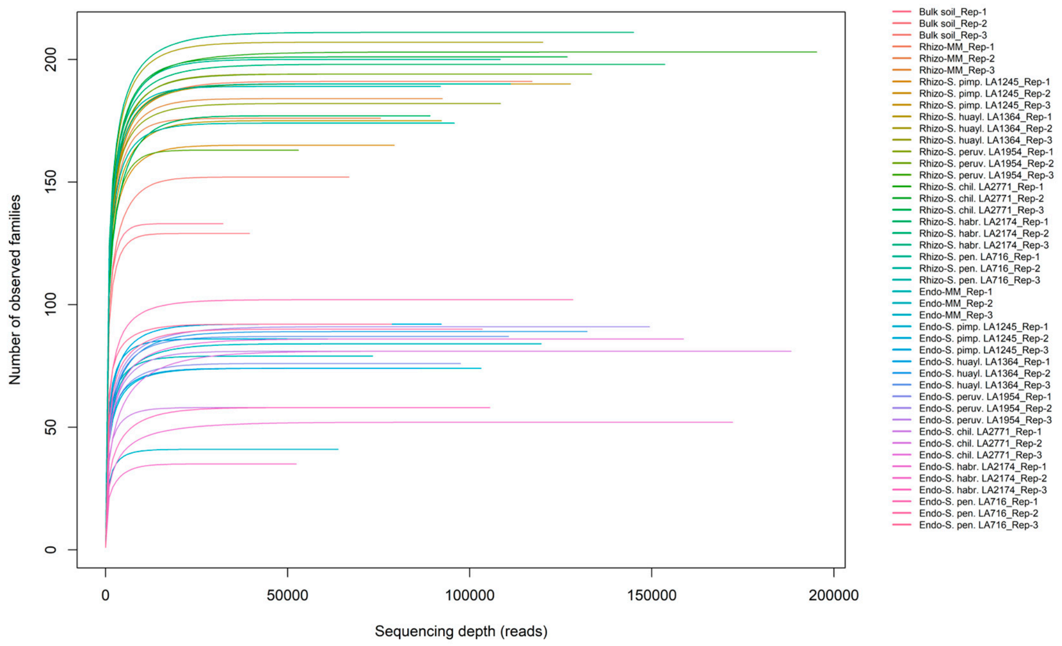

Figure S1. Rarefaction curves for bulk soil, rhizosphere, and endosphere samples, showing the number of observed families plotted against sequencing depth.

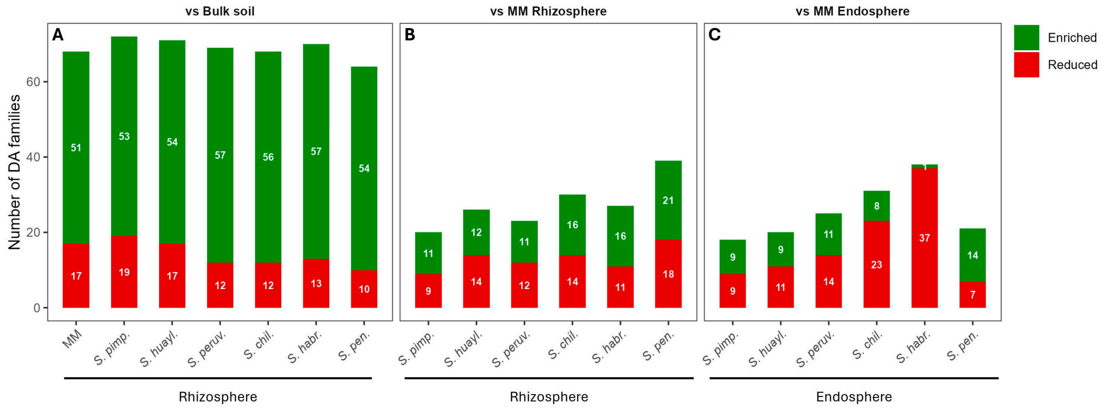

Figure S2. Number of differentially abundant (DA) bacterial families identified by MaAsLin2 using a significance threshold of $q < 0.1$. **(A)** Rhizosphere of each genotype versus bulk soil. **(B)** Rhizosphere of each wild relative versus Moneymaker rhizosphere. **(C)** Endosphere of each wild relative versus Moneymaker endosphere. Numbers within bars indicate the total DA families per category.
